# The effects of Rosa damascene aromatherapy on mood and sleep: a systematic review and meta-analysis

**DOI:** 10.3389/fpubh.2025.1646592

**Published:** 2025-11-04

**Authors:** Shuqi Xu, Xinhua Shen, Liang Xu, Liang Xue, Ping Wu, Shiliang Wang, Xuqiang Hu

**Affiliations:** Department of Psychiatry, Huzhou Third Municipal Hospital, The Affiliated Hospital of Huzhou University, Huzhou, Zhejiang, China

**Keywords:** Rosa damascene aromatherapy, anxiety, depressive symptoms, sleep, systematic review and meta-analysis

## Abstract

**Background and purpose:**

Individuals under stress may experience a range of negative emotions and sleep disturbances. There is preliminary evidence that Rosa damascene (RD) aromatherapy is effective in improving symptoms such as negative mood and sleep. The objective of this study was to assess the effects of RD aromatherapy on anxiety, anxiety-related hemodynamic changes, depression, stress, and sleep quality, while also exploring potential moderating factors that could influence the outcome measures.

**Methods:**

Systematic review and meta-analysis of existing randomized controlled trials on RD aromatherapy in the treatment of mood and sleep. A systematic literature search was conducted across PubMed, Web of Science, EMBASE, CINAHL, and the Cochrane Central Register of Controlled Trials. We identified 28 randomized controlled trials that were pooled using a random-effects meta-analysis.

**Results:**

The meta-analysis demonstrated that RD aromatherapy significantly alleviated anxiety symptoms (SMD = −1.31; 95% CI, −1.74 to-0.88; *p* < 0.001), reduced mean arterial pressure (MAP) (SMD = −0.33; 95% CI, −0.64 to-0.02; *p* = 0.038), and mitigated stress symptoms (SMD = −0.76; 95% CI, −1.07 to −0.44; *p* < 0.001), while also improving sleep quality (SMD = −2.10; 95% CI, −3.54 to −0.66; *p* = 0.004). The effects on depressive symptoms and pulse rate (PR) were minimal (*p* > 0.1).

**Conclusion:**

Our findings suggest that RD aromatherapy can effectively reduce anxiety, improve related hemodynamic parameters, and alleviate stress symptoms, while also enhancing sleep quality. However, its effects on depressive symptoms and PR were smaller, indicating a need for larger randomized trials.

**Systematic review registration:**

Identifier CRD42024593400, https://www.crd.york.ac.uk/PROSPERO/view/CRD42024593400.

## 1 Introduction

With the development of society and the acceleration of life pace, individuals are increasingly exposed to various acute or chronic stressors, which may lead to a range of negative emotions and sleep disturbances (1). Anxiety is defined as an unpleasant experience arising from exposure to perceived or real threats (2), making it one of the most common psychological disorders. Globally, the annual prevalence of anxiety disorders ranges from 2.4 to 29.8%, with a point prevalence of 7.3% (3), while subthreshold anxiety cases are even more common (4). Physiological responses to anxiety can include symptoms such as dyspnea, tachycardia, sweating, tremors, and elevated blood pressure. Prolonged anxiety can compromise the immune system, disrupt fluid and electrolyte balance, and may even result in heightened inflammatory responses, imbalances in protein degradation, and other adverse effects (5, 6). These effects can further increase negative self-perception (7). Numerous studies indicate a high comorbidity between anxiety and depression (8, 9). Patients with anxiety disorders often exhibit depressive symptoms and are frequently accompanied by sleep disturbances (10).

Currently, pharmacological treatments and psychological interventions are the most common approaches for managing anxiety symptoms. However, in clinical practice, patient adherence to medication therapies tends to be poor. For instance, the efficacy of antidepressants and nitrogen-containing heterocyclic ketone drugs may exhibit delayed onset (11), while benzodiazepines and pregabalin can lead to adverse effects such as neurotoxicity, addiction, and tolerance (12). Although cognitive-behavioral therapy and supportive psychotherapy have been proven to be effective (13), they often require significant time and resources. Therefore, there is a need for safer and more effective therapies, particularly targeting subthreshold symptoms of anxiety.

Aromatherapy has been widely used in many healthcare institutions and services as a complementary or alternative therapy for regulating mood and sleep (14). This therapy employs natural plant extracts and other chemical components to stimulate olfactory receptors in the olfactory bulb, transmitting signals to the limbic system to promote the release of various neurotransmitters, such as enkephalins, endorphins, serotonin, and norepinephrine, thereby regulating mood (15). Among the various essential oils available, Damascus rose oil has garnered significant attention due to its unique composition and therapeutic properties. The principal aromatic constituents of Damascus rose oil include geraniol, nerol, phenylethyl alcohol, and their esters (16). Both geraniol and phenylethyl alcohol have been shown to alleviate stress and anxiety, exerting positive effects on the central nervous system (17). Additionally, the potential bioactive compounds present in its composition, such as Methoxymaenin A, Isoquercitrin, Afzelin, Cyanidin-3-O-*β*-glucoside, Quercetin-gentioside, and Damarenone (18), exhibit antioxidant and anti-inflammatory properties, with some compounds positively affecting the cardiovascular and immune systems (19–21).

Clinical research indicates that Rosa damascene (RD) aromatherapy effectively alleviates anxiety and depressive moods in healthy individuals (22) or those experiencing stressors related to childbirth (23, 24), surgery (17, 25), burns (6), and severe illnesses (26), while also improving sleep quality (1, 27). Furthermore, studies have demonstrated the efficacy of Damascus rose oil in reducing work-related stress (28–30). However, existing systematic reviews and meta-analyses concerning Damascus rose oil primarily focus on single populations or specific symptoms. A meta-analysis published in 2022 concentrated solely on pain and anxiety symptoms among burn patients (31), while another meta-analysis examined evidence regarding somatic symptoms in menstruating populations (32). These results reveal significant heterogeneity in the overall anxiolytic effects; however, the potential sources of this heterogeneity, such as participant demographics or intervention characteristics, have yet to be fully determined. The aim of this study is to evaluate the overall impact of RD aromatherapy on anxiety and anxiety-related hemodynamic changes, depression, stress, and sleep quality. Concurrently, we will further categorize the included randomized controlled trials based on their characteristics (such as age, intervention duration, etc.) to investigate potential moderating factors influencing the outcome measures.

## 2 Methods

### 2.1 Search strategy

A systematic literature search of PubMed, Web of Science, EMBASE, CINAHL, and Cochrane Central Register of Controlled Trials was conducted from inception to September 22, 2024, and the search was updated on May 31, 2025. The search string included a combination of synonyms for Rosa damascena, Aromatherapy, and randomized controlled trials (Appendix Table 1). No restrictions were imposed on the outcome measures of interventions during the retrieval of relevant literature. The reference lists of the retrieved literature were further searched to identify any relevant gray literature.

### 2.2 Eligibility criteria

#### 2.2.1 Inclusion criteria

The current meta-analysis followed the Preferred Reporting Items for Systematic Reviews and Meta-analyses (PRISMA) checklist (33). The inclusion criteria for eligible studies were as follows: (1) Population: Clinical trials involving human participants of any age, gender, and health status; (2) Intervention: Any form or formulation of rose oil, rose extract, or other rose-derived therapeutic products; (3) Control: No intervention, standard or routine care, or placebo; (4) Outcomes: The primary outcomes included emotional parameters such as anxiety, depression, and stress symptoms, as well as sleep symptoms, assessed using validated or standardized measurement tools. Secondary outcomes were physiological parameters related to anxiety, including blood pressure, heart rate, or blood oxygen saturation. Specific outcomes for searching the relevant literature were not limited; (5) Study Design: Randomized controlled trials (RCTs). Only manuscripts written in English were included.

#### 2.2.2 Exclusion criteria

We excluded trials that used blended aromatherapy or combined therapies as interventions. Studies lacking essential data were excluded from both the qualitative and quantitative synthesis. Animal studies and *in vitro* research were also excluded.

### 2.3 Literature quality evaluation

We used the Cochrane risk of bias assessment tool (34) to assess the RCTs’ methodological quality, risk of bias in selection, performance, detection, attrition, reporting, and other factors. Two independent reviewers assigned a judgment of high, low, or unclear risk of bias for each of these six domains and then provided a summary assessment for the risk of bias for each study. No study was excluded as a result of findings from the risk of bias assessment.

### 2.4 Statistical method

Statistical analyses were performed with STATA version 15.0 software. Standardized mean differences (SMDs) were calculated for the pooled effects. All estimations are presented with their 95% confidence intervals (95% CIs). All pooled outcome measures were determined using random-effects models. The magnitude of heterogeneity among the included studies was assessed using the chi-squared test (Chi^2^) and I-squared statistic (*I*^2^). For the Chi^2^ test, a Cochran’s Q *p* value of <0.10 was considered significant. An *I*^2^ value of more than 75% was considered to indicate a high degree of heterogeneity, 50–75% was moderate, and 25–50% was a low degree of heterogeneity (35). Sensitivity was examined by assessing the impact of a single study on the pooled overall effect, by omitting one study in turn. Publication bias was evaluated using Egger’s test, and *p* > 0.05 represented the absence of publication bias.

## 3 Results

### 3.1 Study selection

**Figure 1:**
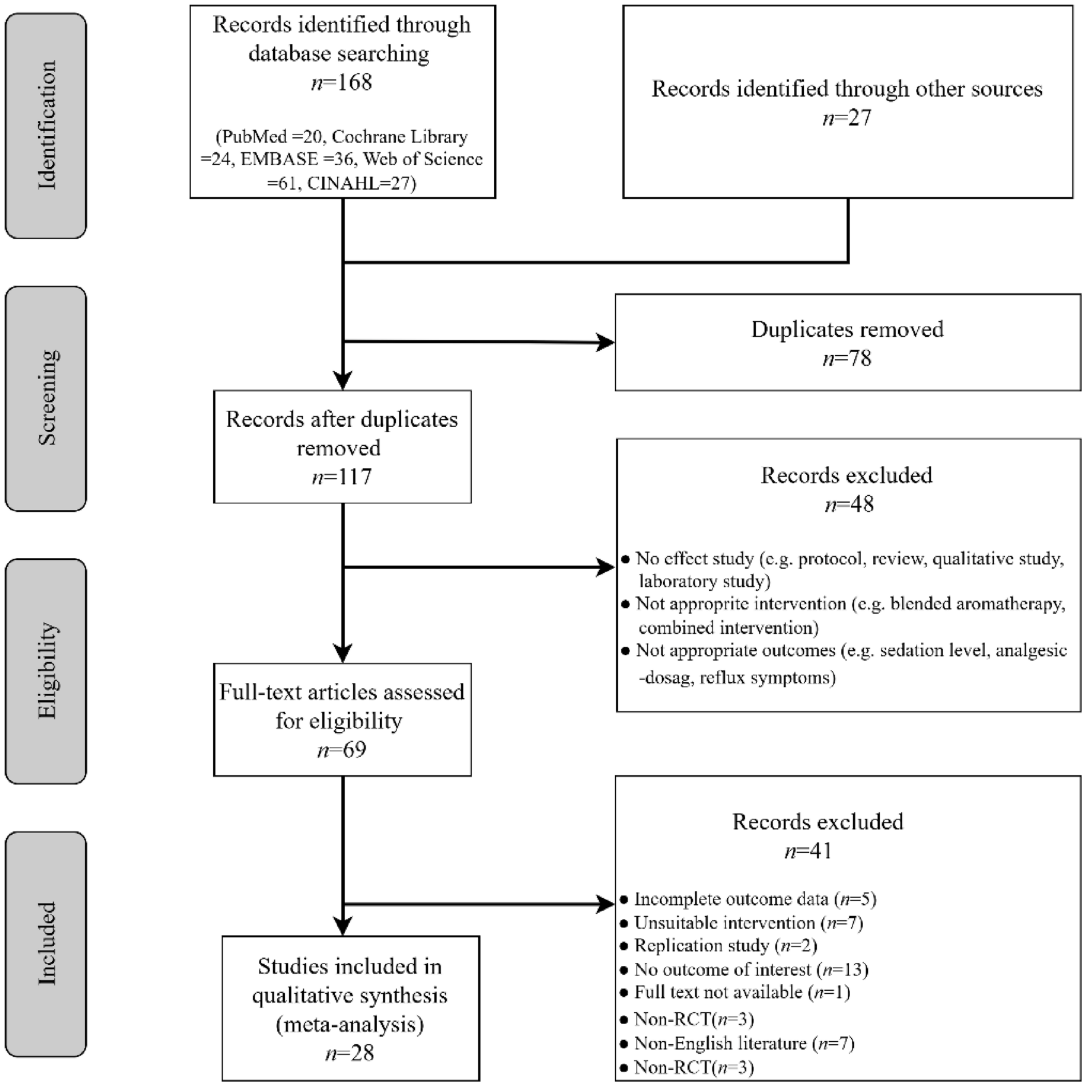
PRISMA flow chart of study selection.

Our literature database search yielded 168 records, and an additional search yielded 27 more records. After removing duplicates, 117 records remained. Of those, 48 records were excluded after screening titles and abstracts. Full reports of 69 publications were acquired, and 41 publications were further excluded for various reasons (see Figure 1). As a result of the eligibility check, 28 articles were finally included. For a further description of our screening process, see the PRISMA study flow diagram (Figure 1).

### 3.2 Study characteristics

Among the 28 included studies, there was one trial each from Thailand, Turkey, India, and China, with the remaining 24 conducted in Iran. The study populations included healthy individuals, patients undergoing surgery or invasive procedures, hemodialysis patients, burn patients, cancer patients, cardiovascular disease patients, pregnant women, premature infants, and healthcare workers under stress. In one trial, subjects received RD aromatherapy via the transdermal route, one used inhalation combined with footbath, and the remaining 26 employed inhalation administration. Control group subjects received standard or conventional treatment, placebo, or no additional intervention. The primary outcomes measured were anxiety, depression, and sleep quality, while secondary outcomes included anxiety-related hemodynamic parameters. The dosage, intervention duration, and treatment regimen for the experimental groups are detailed in Table 1.

**Table 1:** Study characteristics.

Reference	Country	Sample type	*N* (each group)	Age (years, mean)	Method of Administration	Dosage/duration	Control group type	Outcome measure
Hongratanaworakit, (22)	Thailand	Healthy people	20, 20	19.35	Transdermal	1 mL, Apply for 5 min and leave for 20 min	Pure sweet almond oil	PR, BOS
Hajibagheri et al., (4)	Iran	Cardiac patients	30, 30	61.40, 63.90	Inhalation	3 drops each night for 8 h	Usual sleep care	PSQI
Kheirkhah et al., (51)	Iran	Nulliparous women	36, 36	-	Inhalation and Footbath	10 min	Routine care of the delivery room	VAS-A
Babaii et al., (52)	Iran	Patients before cardiac catheterization	30, 30	53.63, 56.96	Inhalation	3 drops, 18 min	Routinely rested	STAI
Dehkordi et al., (53)	Iran	Hemodialysis patients	28, 28	58.90, 58.20	Inhalation	3 drops, 60 min for 1 month	Usual care	DASS-21
Hamdamian et al., (23)	Iran	Nulliparous women	55, 55	25.87, 26.24	Inhalation	2 drops (0.8 mL), 10 min	Normal saline	STAI
Dagli et al., (54)	Turkey	Patients undergoing septorhinoplasty/rhinoplasty	33, 33, 33	28.61, 27.06, 26.58	Inhalation	15 min	No additional intervention; A mixture of ethyl alcohol	STAI-S, MAP, PR
Daneshpajooh et al., (55)	Iran	Burn patients	33, 33	44.10, 40.12	Inhalation	5 drops, 20 min for 3 days	Usual care	BSPAS
Fazlollahpour-Rokni et al., (56)	Iran	Patients undergoing coronary artery bypass graft surgery	32,33	62.30, 63.09	Inhalation	3 drops, 10 min	No additional intervention	STAI
Heydarirad et al., (57)	Iran	Cancer patients	15, 15, 15	47.60, 50.00, 50.20	Inhalation	5 drops, 20 min for 2 weeks	No additional intervention	PSQI
Premkumar et al., (58)	India	Orthodontic Patients	24, 24	20.54	Inhalation	15 min	Plain water	PR, SBP, DBP
Abbasijahromi et al., (59)	Iran	Healthy singleton parturient undergoing cesarean delivery	30, 30	27.60, 29.73	Inhalation	3 drops, 30 min	Normal saline	STAI
Babatabar Darzi et al., (60)	Iran	Patients after open-heart surgery	40, 40, 40	60.50, 62.27, 57.50	Inhalation	3 drops, 15 min	Usual care or Placebo	STAI-S
Sadeghi et al., (6)	Iran	Burn patients	40, 37, 40	37.20, 36.98, 34.40	Inhalation	6 drops, 60 min	No additional intervention or Distilled water	STAI
Farsi et al., (61)	Iran	Nurses in the emergency department	30, 30	29.40, 28.73	Inhalation	2 drops, 10 min	Distilled water	NSS
Jodaki et al., (62)	Iran	Cardiac patients	30, 30	62.80, 61.50	Inhalation	5 drops each night for 8 h, 3 days	Distilled water	SMHSQ, STAI-S
Jirdehi et al., (63)	Iran	Endoscopy patients	35, 35	43.44	Inhalation	2 drops, 30 min	Unscented soybean oil	STAI-S
Bahadori et al., (30)	Iran	Operating Room Nurses	30, 30	31.23, 33.20	Inhalation	2 drops, 10 min	Normal saline	STAI, JSQ
Bikmoradi et al., (64)	Iran	Patients undergoing coronary angiography	49, 49	59.47, 62.62	Inhalation	5 drops, 20 min	Distilled water	DASS-21, MAP, PR, SBP, DBP, BOS
Farzaneh et al., (25)	Iran	Patients undergoing Percutaneous Nephrolithotomy	19, 19	49.21, 47.74	Inhalation	3 drops, 30 min	Distilled water	STAI
Haddadi et al., (26)	Iran	Patients with myocardial infarction	40, 40	-	Inhalation	3 drops, 20–30 min, 3 times a day with an interval of 30 min between inhalations for 3 days	Sesame oil	STAI-S
Mahdood et al., (29)	Iran	Operating Room Personnel During the COVID-19 Pandemic	40, 40	31.52, 33.05	Inhalation	2 drops, 10 min; 5 drops, 8 h for 30 consecutive nights;	Paraffin oil	STAI-S, PSQI
Emadikhalaf et al., (28)	Iran	Nurses	40, 39	35.90, 36.49	Inhalation	0.5 mL, 2 h a day within a 4-week period	Sesame oil	NSS
Mokhtari et al., (1)	Iran	Burn patients	30, 30	35.4	Inhalation	5 drops each night for 8 h, 3 days	Distilled water	SMHSQ, STAI-S
Askarinia et al., (65)	Iran	Preterm infants	25, 25	30.40 weeks, 30.48 weeks	Inhalation	2 drops, 5 min before venipuncture to 2 min after it	Placebo	PR, BOS
Bahrami et al., (17)	Iran	Emergency orthopedic surgery patients	30, 30	42.17, 46.87	Inhalation	3 drops, in the second and third hours, each time with three more drops of essential oil	Placebo	VAS-A
Hosseini et al., (27)	Iran	Primiparous Women	37, 37	27.32, 26.00	Inhalation	1 ml, inhaling deeply 10 times, overnight	Distilled water	EPDS, PSQI
Li et al., (24)	China	Primiparous Women	26, 24, 26	28.19, 27.04, 27.46	Inhalation	0.1 ml/h at least 30 min	Usual care or Normal saline	VAS-A

PR, Pulse Rate; BOS, Blood Oxygen Saturation; PSQI, Pittsburgh Sleep Quality Index; VAS, Visual Analogue Scales; VAS-A, Visual Analog Scale For Anxiety; STAI, State–Trait Anxiety Inventory; DASS, Depression Anxiety Stress Scales; STAI-S, State–Trait Anxiety Inventory- State Questionnaire of Spielberger; MAP, Mean Arterial Pressure; BSPAS, Burn Specific Pain Anxiety Scale; SBP, Systolic Blood Pressure; DBP, Diastolic Blood Pressure; NSS, Nursing Stress Scale; SMHSQ, St Mary’s Hospital Sleep Quality Questionnaire; JSQ, Job Stress Questionnaire; EPDS, Edinburgh Postnatal Depression Scale.

### 3.3 Risk of bias assessment

In all studies, there was a low risk of bias for most items (Appendix Figure 1), except for the presence of detection bias due to the lack of a double-blind design in 13 studies. All articles included were described as randomized controlled trials. Allocation concealment was assessed as high risk in three trials (11%) because researchers were able to predict group assignments, and as unclear risk in four trials (14%). Nine trials (32%) were considered to have a high risk of performance bias because the nature of the aromatherapy intervention made it easily identifiable by the participants. The majority of the trials (89%) were judged to have a low risk of attrition bias, while three trials (11%) were assessed as having an unclear risk of attrition bias. Eight trials (29%) were considered to have a high risk of other bias due to significant confounding factors in the study design.

### 3.4 Psychological distress, sleep symptom outcome measures

The results for anxiety symptoms included overall anxiety, state anxiety, and trait anxiety. Overall anxiety was assessed using various measurement tools, including the Depression Anxiety Stress Scale-21 (DASS-21, 36), the Visual Analogue Scale for Anxiety (VAS-A) (37), the State–Trait Anxiety Inventory total score (STAI total) (38), the State–Trait Anxiety Inventory-State (STAI-S) (38), and the Burn Specific Pain Anxiety Scale (BSPAS) (39). State anxiety was evaluated with the VAS-A, STAI-S, and BSPAS, while trait anxiety was assessed with the State–Trait Anxiety Inventory-Trait (STAI-T) (38). Depressive symptoms were evaluated using the DASS-21 and the Edinburgh Postnatal Depression Scale (EPDS) (40). Stress symptoms were assessed using the DASS-21, the Nursing Stress Scale (NSS) (41), and the Job Stress Questionnaire (JSQ) (42). Sleep symptoms were evaluated using the Pittsburgh Sleep Quality Index (PSQI) (43) and the St. Mary’s Hospital Sleep Questionnaire (SMHSQ) (44). Additionally, the study collected hemodynamic parameters, including systolic blood pressure (SBP), diastolic blood pressure (DBP), mean arterial pressure (MAP), pulse rate (PR), and blood oxygen saturation (BOS).

### 3.5 Effects on anxiety symptom

**Figure 2:**
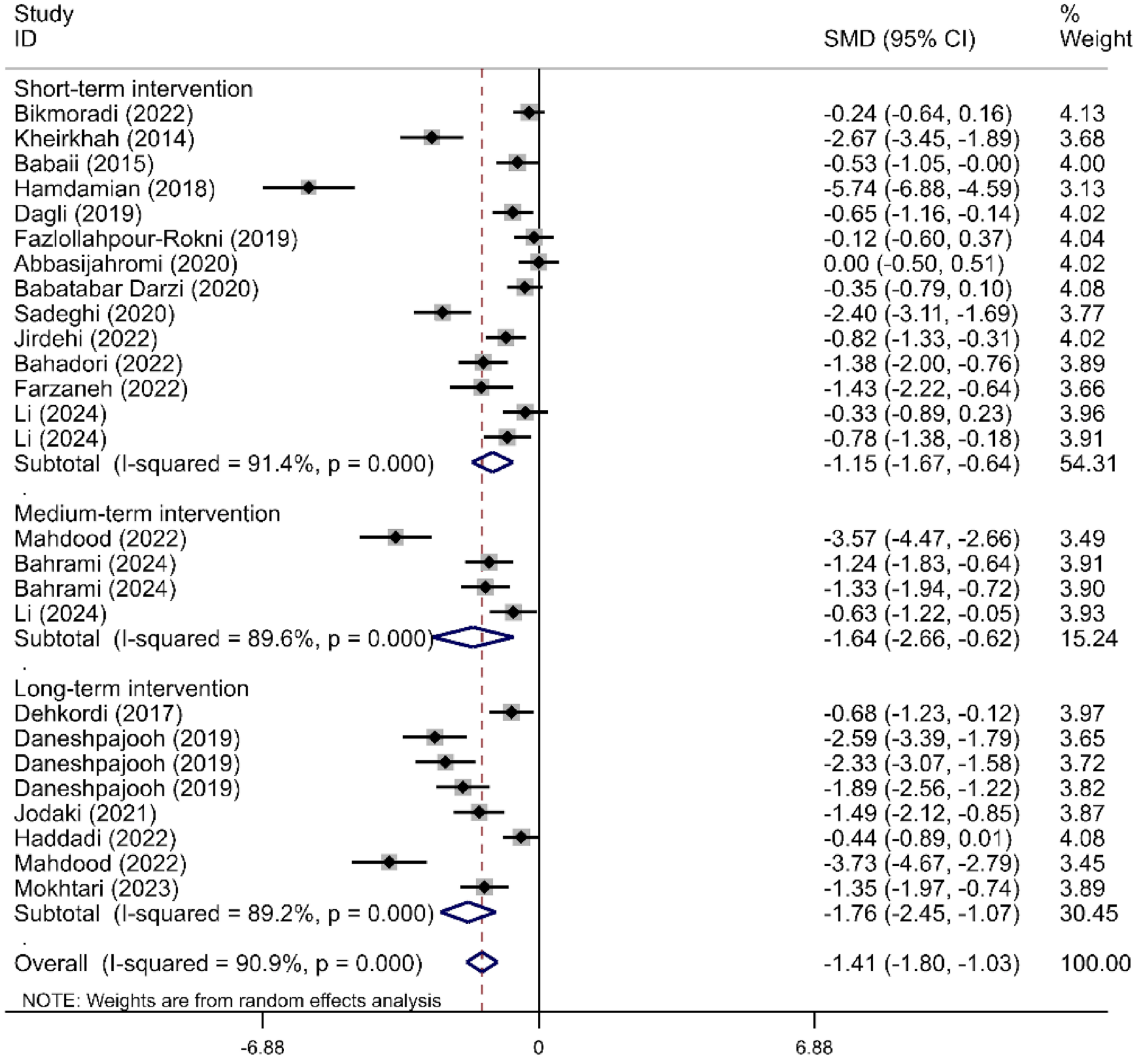
Forest plot comparing RD aromatherapy and the control group, stratified by treatment duration. Outcome: overall anxiety scores.

**Figure 3:**
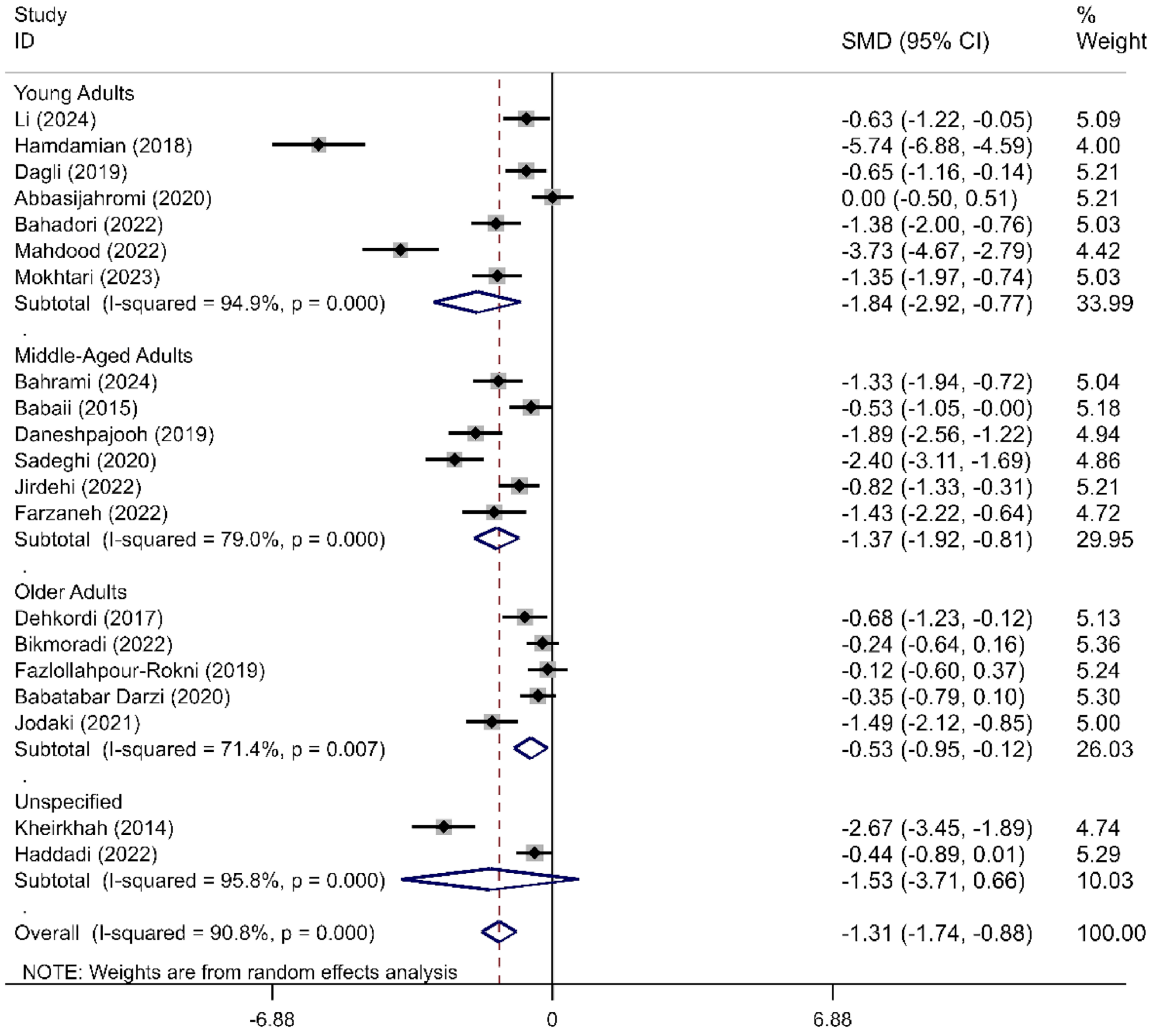
Forest plot comparing RD aromatherapy and the control group, stratified by age. Outcome: overall anxiety scores.

**Figure 4:**
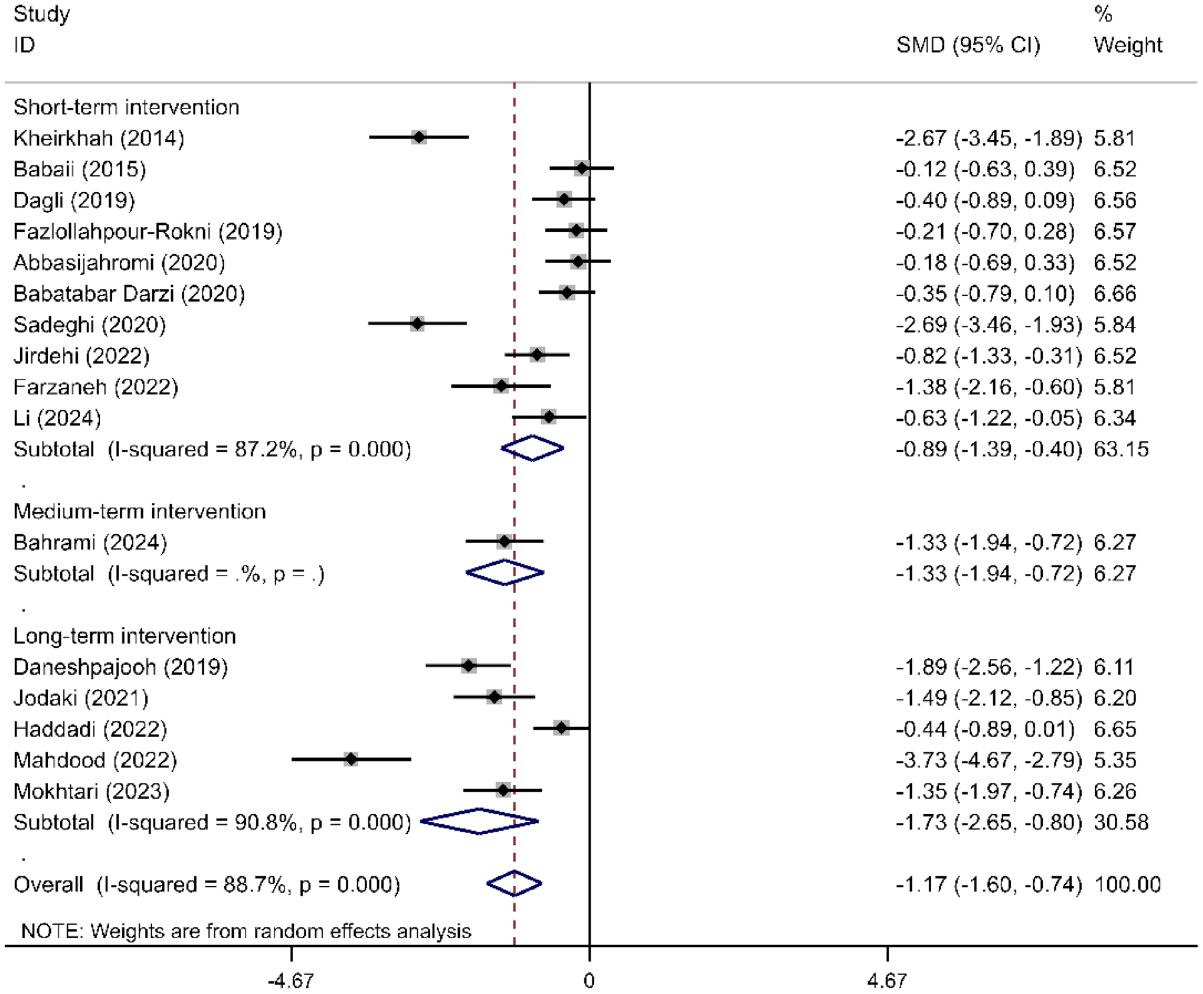
Forest plot comparing RD aromatherapy and the control group, stratified by treatment duration. Outcome: state anxiety.

**Figure 5:**
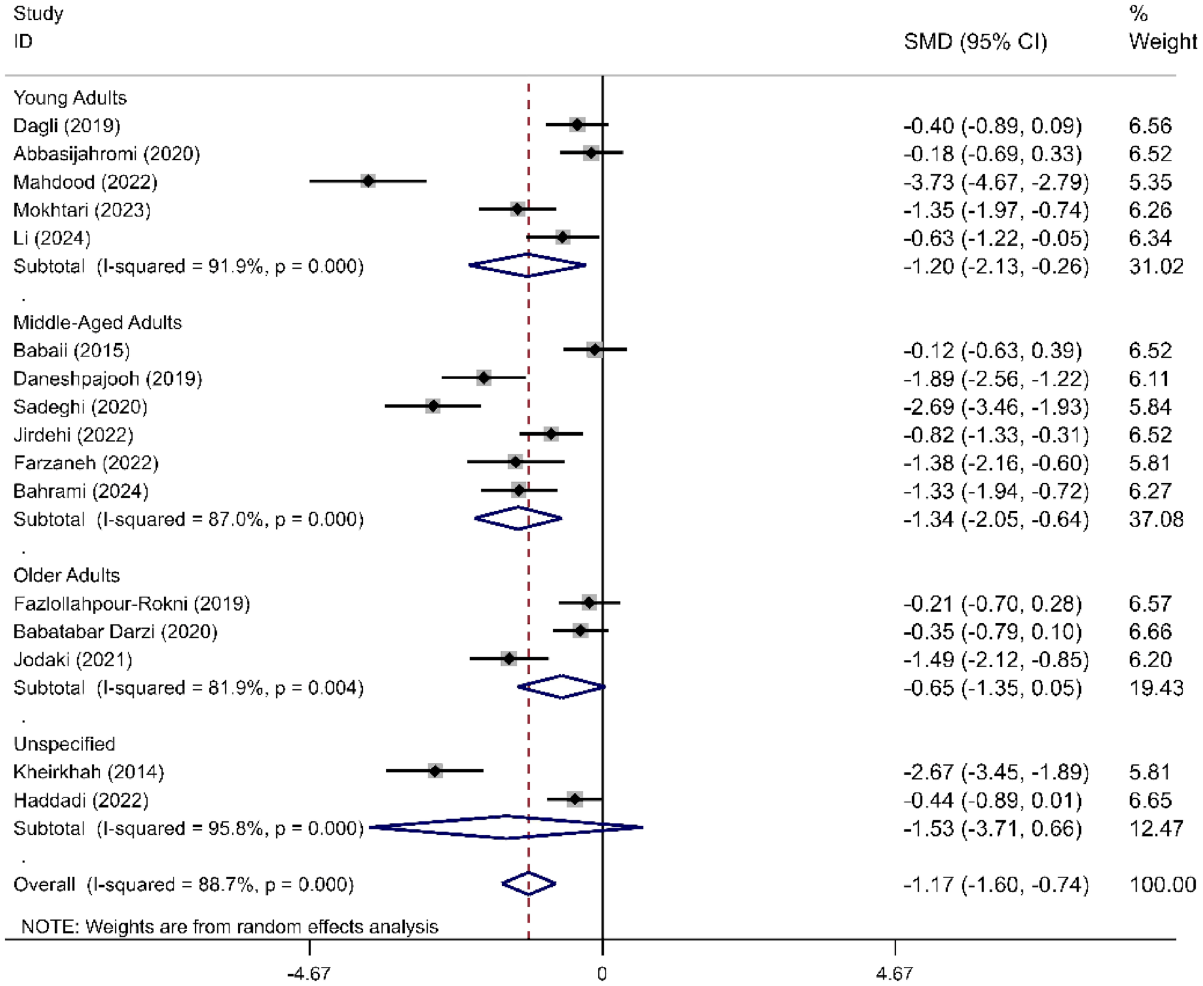
Forest plot comparing RD aromatherapy and the control group, stratified by age. Outcome: state anxiety.

**Figure 6:**
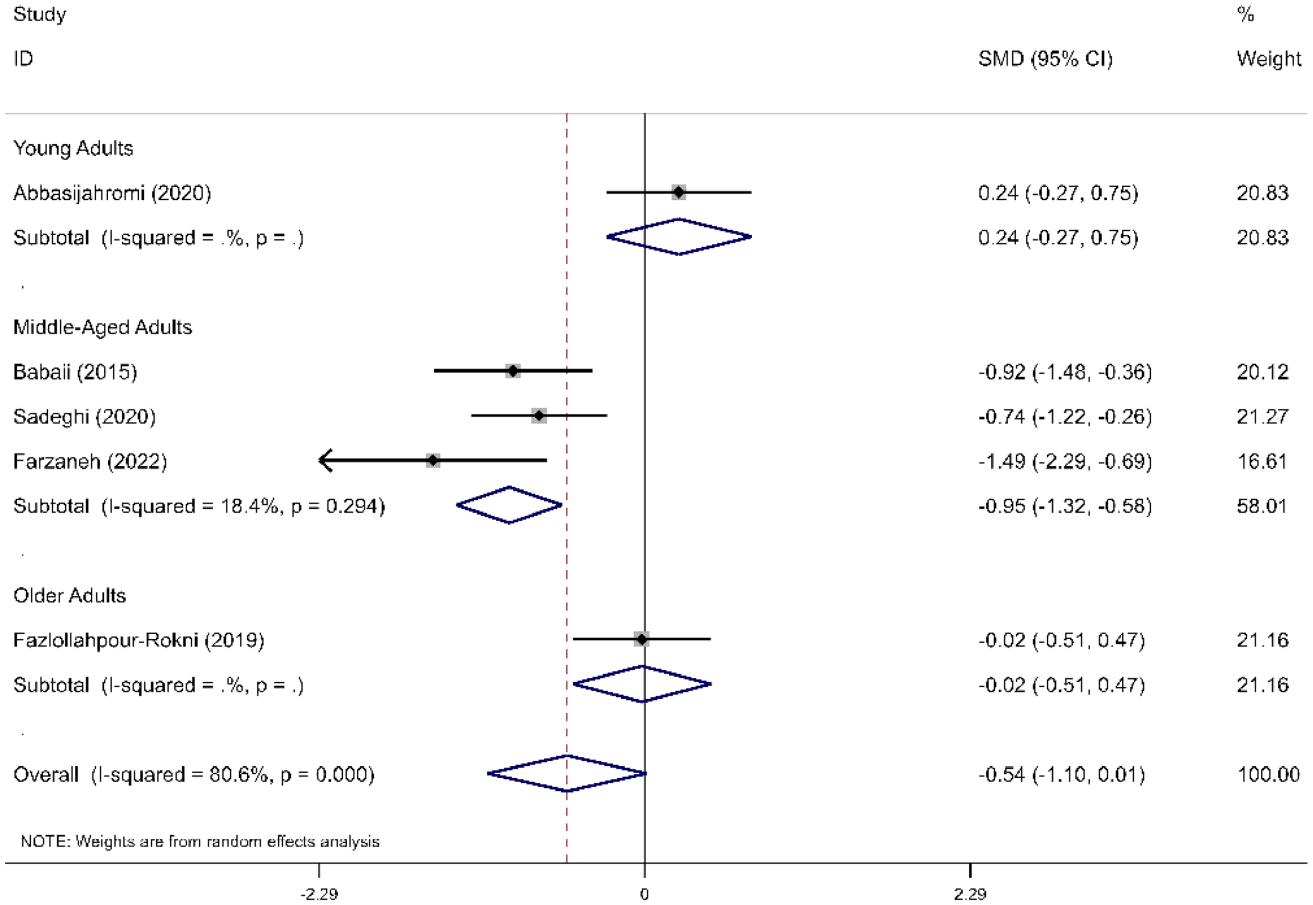
Forest plot comparing RD aromatherapy and the control group, stratified by age. Outcome: trait anxiety.

We determined the pooled effect size of RD aromatherapy on anxiety symptom and compared it to the anxiety symptom of the control group in a random effects model. The effect size showed significant difference between the two groups’ overall anxiety scores (SMD = -1.31; 95% CI, −1.74 to −0.88; *p*<0.001). The test for heterogeneity among the individual studies was significant (*I*^2^ = 90.8%, *p*<0.001). In the subgroup analysis based on the duration of intervention, RD aromatherapy showed significantly greater efficacy compared to the control group across different intervention durations (SMD short = −1.15, *p* < 0.001; SMD medium = −1.64, *p* = 0.002; SMD long = −1.76, *p* < 0.001) (Figure 2). The results of the subgroup analysis based on age showed that RD aromatherapy was effective in reducing overall anxiety compared to the control group across different age groups (SMD young = −1.84, *p* = 0.001; SMD middle = −1.37, *p* < 0.001; SMD older = −0.53, *p* = 0.012) (Figure 3). RD aromatherapy demonstrated significantly greater overall efficacy for state anxiety compared to the control group (SMD = -1.17; 95% CI, −1.60 to −0.74; *p*<0.001). The test for heterogeneity among the individual studies was significant (*I*^2^ = 88.7%, *p*<0.001). Subgroup analysis based on the duration of intervention showed that the intervention group outperformed the control group across different intervention durations (SMD short = −0.89, *p* < 0.001; SMD medium = −1.33, *p* < 0.001; SMD long = −1.73, *p* < 0.001) (Figure 4). Subgroup analysis by age revealed that RD aromatherapy was more effective for state anxiety in the young and middle-aged groups, whereas its efficacy was not significant in the older group (SMD young = −1.20, *p* = 0.010; SMD middle = −1.34, *p* < 0.001; SMD older = −0.65, *p* = 0.069) (Figure 5). Our results showed that RD aromatherapy had no significant effect on trait anxiety (SMD = −0.55; 95% CI, −1.10 to −0.01; *p* = 0.056). The heterogeneity test revealed substantial heterogeneity (*I*^2^ = 80.6%, *p* < 0.001). Subgroup analysis indicated that the intervention was more effective in reducing trait anxiety in the middle-aged group (SMD = −0.95, *p* < 0.001) (Figure 6).

### 3.6 Effects on depression symptoms

For depressive symptoms, there was no statistically significant difference between RD aromatherapy and the control group (SMD = 0.07; 95% CI, −0.67 to 0.81; *p* = 0.848). Heterogeneity was significant (*I*^2^ = 77.0%, *p* = 0.037). Only two studies were included, and we did not perform subgroup analysis.

### 3.7 Effects on stress symptom

**Figure 7:**
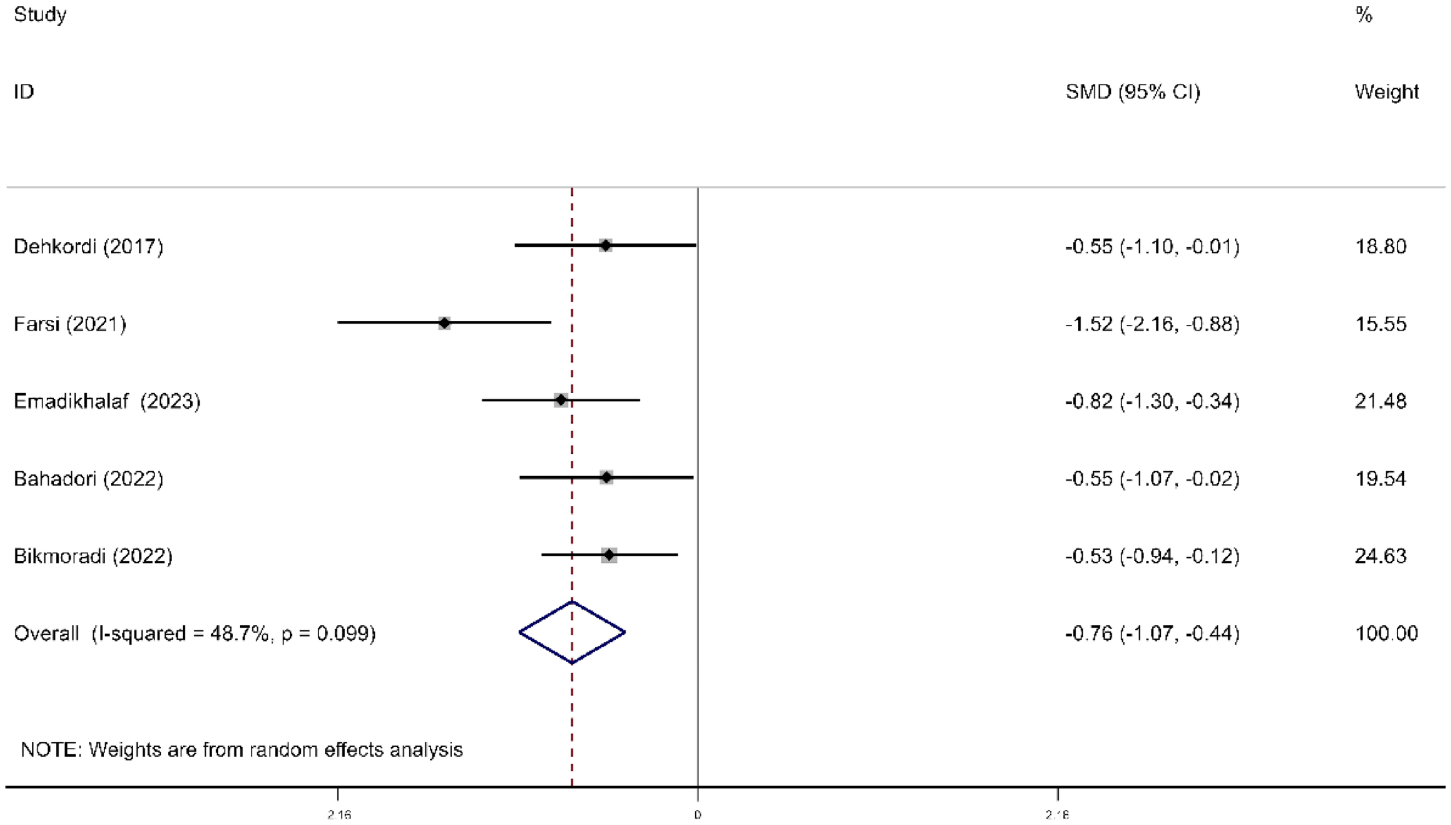
Forest plot comparing RD aromatherapy and the control group. Outcome: stress symptom.

For stress symptoms, RD aromatherapy demonstrated significantly greater efficacy compared to the control group (SMD = -0.76; 95% CI, −1.07 to −0.44; *p* < 0.001). Heterogeneity was low (*I*^2^ = 48.7%, *p* = 0.099) (Figure 7).

### 3.8 Effects on sleep symptom

**Figure 8:**
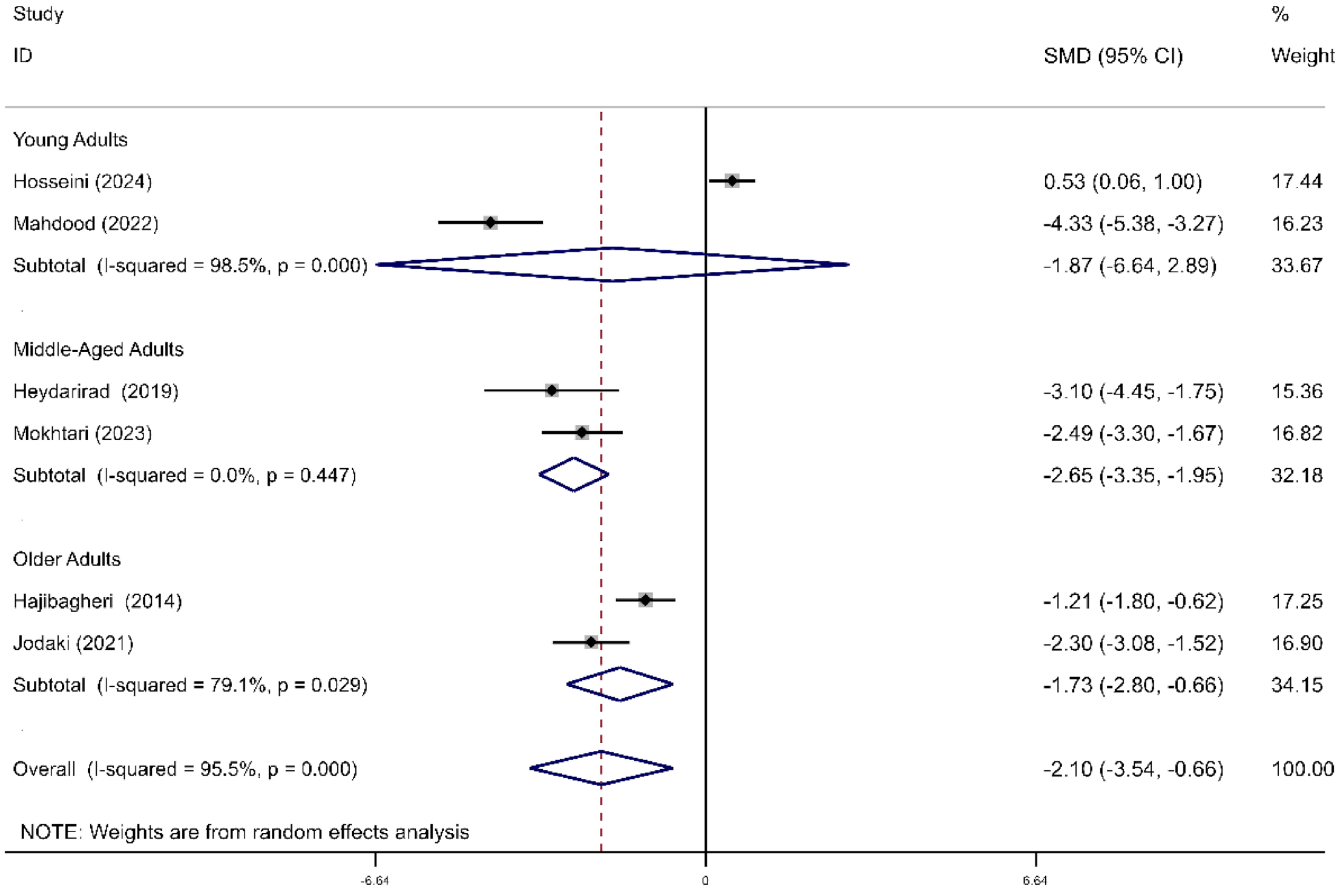
Forest plot comparing RD aromatherapy and the control group, stratified by age. Outcome: sleep symptom.

Compared to the control group, RD aromatherapy significantly improved overall sleep symptoms (SMD = -2.10, 95% CI, −3.54 to −0.66, *p* = 0.004). Heterogeneity was significant (*I*^2^ = 95.5%, *p* < 0.001). Subgroup analysis showed that the intervention group demonstrated significant improvements in overall sleep symptoms in middle-aged and older adults, while the effect was not significant in the young group (SMD young = −1.87, *p* = 0.441; SMD middle = −2.65, *p* < 0.001; SMD older = −1.73, *p* = 0.002) (Figure 8).

### 3.9 Effects on hemodynamic parameters

The results showed that RD aromatherapy significantly improved MAP (SMD = −0.33, 95% CI, −0.64 to −0.02, *p* = 0.038), while no significant effects were observed on SBP, DBP, or PR (*p* > 0.1).

### 3.10 Publication bias and sensitivity analysis

Egger’s test was performed to evaluate the publication bias of the included studies. The results indicated the presence of publication bias for state anxiety outcomes (*p* < 0.01), while no publication bias was detected for trait anxiety, stress symptoms, or sleep symptoms (*p* = 0.247; *p* = 0.189; *p* = 0.016) (Appendix Figures 2–5). To assess the stability of the results of the studies, a sensitivity analysis was performed by successively omitting each individual study. The study by Hosseini (27) on sleep symptoms yielded negative results, making it highly specific in the sensitivity analysis; thus, the findings from this study should be interpreted with caution. There was no alteration in the results for other outcomes, indicating that our findings were statistically reliable and robust. Details of the sensitivity analysis are provided in Appendix Figures 6–10.

## 4 Discussion

In this meta-analysis, we evaluated and synthesized clinical trial evidence regarding the effects of RD aromatherapy on mood, hemodynamic changes, and sleep quality. The results indicate that RD aromatherapy improves overall anxiety across all age groups, regardless of whether the intervention duration is short-term or long-term. Furthermore, we conducted a more detailed classification of anxiety types, distinguishing between state anxiety and trait anxiety. State anxiety refers to the temporary anxiety experienced in response to potential threats, while trait anxiety is the individual’s susceptibility to anxiety (45), a relatively stable characteristic often associated with neurocognitive deficits (46). Although the pooled analysis indicated a significant improvement in state anxiety following RD aromatherapy, the detected publication bias suggests that this effect may be overestimated. The results of the subgroup analysis showed that RD aromatherapy significantly alleviates state anxiety in younger adults, while its effects on the older adults are less pronounced. Improvements in trait anxiety were observed only in the middle-aged group. RD aromatherapy has proven effective in reducing stress, MAP, and sleep symptoms, particularly in the middle-aged and older populations; however, its impact on depressive symptoms and PR was not significant.

Koohpayeh et al. (32) found that RD aromatherapy had no significant effect on menstrual-related anxiety, although their analysis included only three studies with low methodological quality. Conversely, Farzan et al. (47) reported that RD aromatherapy effectively reduced anxiety levels in burn patients, which is consistent with our findings from a broader population analysis. Additionally, this study revealed that RD aromatherapy improves sleep, stress, and certain hemodynamic parameters. The aromatic components of Damascus rose oil, including geraniol and phenethyl alcohol, have been demonstrated to alleviate stress and promote sleep (17). These odor molecules bind to olfactory receptors on the olfactory epithelial cells, generating electrical signals that are transmitted directly to the limbic system of the brain via the olfactory nerve (16). This process helps mitigate excessive amygdala activity, regulate sleep–wake cycles (48), and balance stress responses through the HPA axis (48). It is important to emphasize that some statistical heterogeneity in this meta-analysis was high, likely due to variations in baseline levels of outcome measures, types of study populations (ranging from healthy individuals to critically ill patients), intervention protocols, essential oil compositions, and psychometric scales. These factors may act as moderating variables influencing the outcome measures. The negative results reported by Hosseini (27) regarding sleep symptoms highlight their high specificity in sensitivity analysis; we interpret this as the postpartum stress having a greater impact on sleep quality than what aromatherapy could alleviate. This suggests that effective coping strategies and social support are equally crucial in times of stress (49). RD aromatherapy did not have a significant impact on depression and PR, which may be attributed to an insufficient sample size affecting statistical power. Moreover, we observed that the middle-aged demographic may be a significant beneficiary of RD aromatherapy. The efficacy of aromatherapy may vary based on individual characteristics (50), warranting further research beyond the scope of this report to understand these variations.

### 4.1 Strengths and limitations

The advantage of this meta-analysis lies in its provision of high-quality cumulative evidence from well-designed randomized clinical trials, suggesting that RD aromatherapy may be effective in improving sleep quality and alleviating psychological distress. Unlike previous meta-analyses, this study not only explored these outcomes based on the duration of the intervention but also categorized them according to age groups. The analysis was conducted rigorously in accordance with the Cochrane Handbook (34) and PRISMA guidelines (33). However, several limitations must be addressed. Heterogeneity among studies persisted in some subgroups, indicating the presence of other potential moderating factors arising from differences in intervention protocols (such as the concentration and dosage of essential oils, duration of aromatherapy sessions, and treatment course), sample sizes, population types, and other variables. Future studies with more rigorous designs and standardized protocols are needed to confirm these findings and identify the optimal conditions for its effects. Although the included study populations were clinically diverse, the pronounced geographical imbalance (85.7% of studies originating from Iran) remains a significant limitation. Future investigations across diverse cultural and social settings are warranted to validate the generalizability of these effects. Furthermore, implementing a strict blinding procedure during the intervention was challenging due to the distinctive scent of damascene rose essential oil, which led to unavoidable bias in the results. Future studies should employ more sophisticated designs, such as the use of active placebo controls, to differentiate between physiological and psychological effects. Additionally, we did not obtain sufficient data to determine whether RD aromatherapy improved depressive symptoms and certain hemodynamic parameters in the trial population. The positive effect of RD aromatherapy on sleep symptoms is highly susceptible to the influence of individual studies. Therefore, more homogeneous future research is needed to provide more reliable evidence regarding its sleep-improving effects.

## 5 Conclusion

Our findings indicate that RD aromatherapy can effectively improve anxiety, certain related hemodynamic parameters, and stress symptoms, and may have a positive effect on improving sleep quality. Notably, its simplicity, safety, and low cost render it a viable therapeutic option that can be considered in specific clinical settings. The effects on depressive symptoms and PR demonstrated smaller effect sizes, suggesting the need for more extensive randomized trials. There are indications that middle-aged individuals may be a significant beneficiary group of RD aromatherapy. These results are derived from randomized clinical trials and require further validation.

## Data Availability

The original contributions presented in the study are included in the article/Supplementary material, further inquiries can be directed to the corresponding authors.
