# Supplementary material for: The effects of Rosa damascene aromatherapy on mood and sleep: a systematic review and meta-analysis
Source: Front Public Health. 2025 Nov 4;13:1646592. doi: 10.3389/fpubh.2025.1646592 (PMC12623198; doi:10.3389/fpubh.2025.1646592)

**Appendix** **Table1**

Date: 22 September 2024

Update: 31 May 2025

**Search strategy PubMed**

| **Set** | **Terms** | **Results** |
| --- | --- | --- |
| #1 | "Rosa damascena"[tiab] OR "Damask rose"[tiab] OR "Rose of Damascus"[tiab] OR "Turkish rose"[tiab] OR "Bulgarian rose"[tiab] OR "Isparta rose"[tiab] OR "Persian rose"[tiab] OR "Attar of rose"[tiab] OR "damascene"[tiab] | **406** |
| #2 | "Aromatherapy"[Mesh] OR "Aromatherapies"[tiab] OR " Aroma Therapy"[tiab] OR " Aroma Therapies"[tiab] OR " Therapies, Aroma"[tiab] OR " Therapy, Aroma"[tiab] | **1,173** |
| #3 | (("randomized controlled trial"[pt] OR "Controlled Clinical Trial"[pt] OR "randomized"[tiab] OR "randomised"[tiab] OR "randomization"[tiab] OR "randomisation"[tiab] OR "placebo"[tiab] OR "randomly"[tiab] OR "trial"[tiab] OR "groups"[tiab] OR "Comparative Study"[pt] OR "Controlled Clinical Trial"[pt] OR "Nonrandom"[tiab] OR "Nonrandom"[tiab] OR "nonrandomized"[tiab] OR "non-randomized"[tiab] OR "nonrandomized"[tiab] OR "non-randomised"[tiab] OR "quasi experiment*"[tiab] OR "quasiexperiment*"[tiab] OR "quasirandom*"[tiab] OR "quasi random*"[tiab] OR "quasi control*"[tiab] OR "quasicontrol*"[tiab] OR ("controlled"[tiab] AND ("trial"[tiab] OR "study"[tiab]))) NOT ("animals"[Mesh] NOT "humans"[Mesh])) NOT ("Editorial"[pt] OR "Letter"[pt] OR "case reports"[pt] OR "Comment"[pt]) | **4,656,209** |
| #4 | #1 AND #2 AND #3 | **20** |

**Search strategy EMBASE**

| **Set** | **Terms** | **Results** |
| --- | --- | --- |
| #1 | 'Rosa damascena'/exp OR 'Damask rose':ab,ti OR 'Rosa x damascena':ab,ti OR 'Rose of Damascus':ab,ti OR 'Turkish rose':ab,ti OR 'Bulgarian rose':ab,ti OR ' Isparta rose':ab,ti OR 'Attar of rose':ab,ti OR 'damascene':ab,ti | **716** |
| #2 | 'aromatherapy'/exp OR 'aromatherapies':ab,ti OR 'aroma therapy':ab,ti OR 'aroma therapies':ab,ti OR 'therapies, aroma':ab,ti OR 'therapy, aroma':ab,ti | **3,283** |
| #3 | 'randomized controlled trial'/exp OR 'controlled trial, randomized' OR 'randomised controlled study' OR 'randomised controlled trial' OR 'randomized controlled study' OR 'randomized controlled trial' OR 'trial, randomized controlled' | **1,151,235** |
| #4 | #1 AND #2 AND #3 | **36** |

**Search strategy Cochrane Library**

#1 MeSH descriptor: [Aromatherapy] explode all trees 405

#2 (aromatherapies):ti,ab,kw (Word variations have been searched) 11

#3 (aroma therapy):ti,ab,kw (Word variations have been searched) 204

#4 (aroma therapies):ti,ab,kw (Word variations have been searched) 15

#5 (therapies, aroma):ti,ab,kw (Word variations have been searched) 15

#6 (therapy, aroma):ti,ab,kw (Word variations have been searched) 204

#7 #1 OR #2 OR #3 OR #4 OR #5 OR #6 506

#8 (Rosa damascena):ti,ab,kw (Word variations have been searched) 129

#9 (Damask rose):ti,ab,kw (Word variations have been searched) 54

#10 (Rosa x damascena):ti,ab,kw (Word variations have been searched) 2

#11 (Turkish rose):ti,ab,kw (Word variations have been searched) 3

#12 (Rose of Damascus):ti,ab,kw (Word variations have been searched) 1

#13 (Isparta rose):ti,ab,kw (Word variations have been searched) 1

#14 (Persian rose):ti,ab,kw (Word variations have been searched) 20

#15 (damascene):ti,ab,kw (Word variations have been searched) 42

#16 #8 OR #9 OR #10 OR #11 OR #12 OR #13 OR #14 OR #15 207

**24**Trials matching **"#17 -** **#7 AND #16"**

**Search strategy Web of Science Results：61**

*Refined By Languages: English. Document Types: Article or Clinical Trial.*

((Rosa damascena) OR (Damask rose) OR (Rose of Damascus) OR (Turkish rose) OR (Bulgarian rose) OR (Isparta rose) OR (Persian rose) OR (Attar of rose) OR (damascene)) AND ((Aromatherapy) OR (Aromatherapies) OR (Aroma Therapy) OR (Aroma Therapies) OR (Therapies, Aroma) OR (Therapy, Aroma))

**Search strategy CINAHL(EBSCO) Results：27**

*Qualifications - English; Research papers; Do not include the Pre - CINAHL; Exclude MEDLINE records; Humans; Age group: All Adult Search mode - Boolean logic/phrase*

((Rosa damascena) OR (Damask rose) OR (Rose of Damascus) OR (Turkish rose) OR (Bulgarian rose) OR (Isparta rose) OR (Persian rose) OR (Attar of rose) OR (damascene)) AND ((Aromatherapy) OR (Aromatherapies) OR (Aroma Therapy) OR (Aroma Therapies) OR (Therapies, Aroma) OR (Therapy, Aroma))

**Appendix Figure 1. Quality assessment of RCTs.**


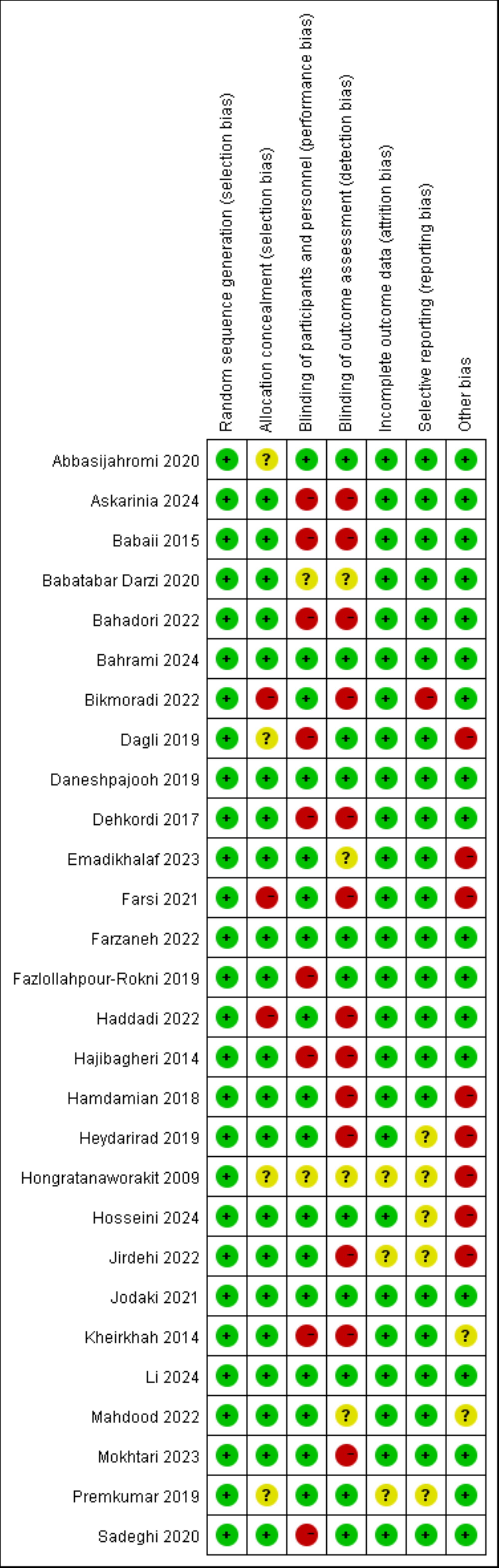
**A**


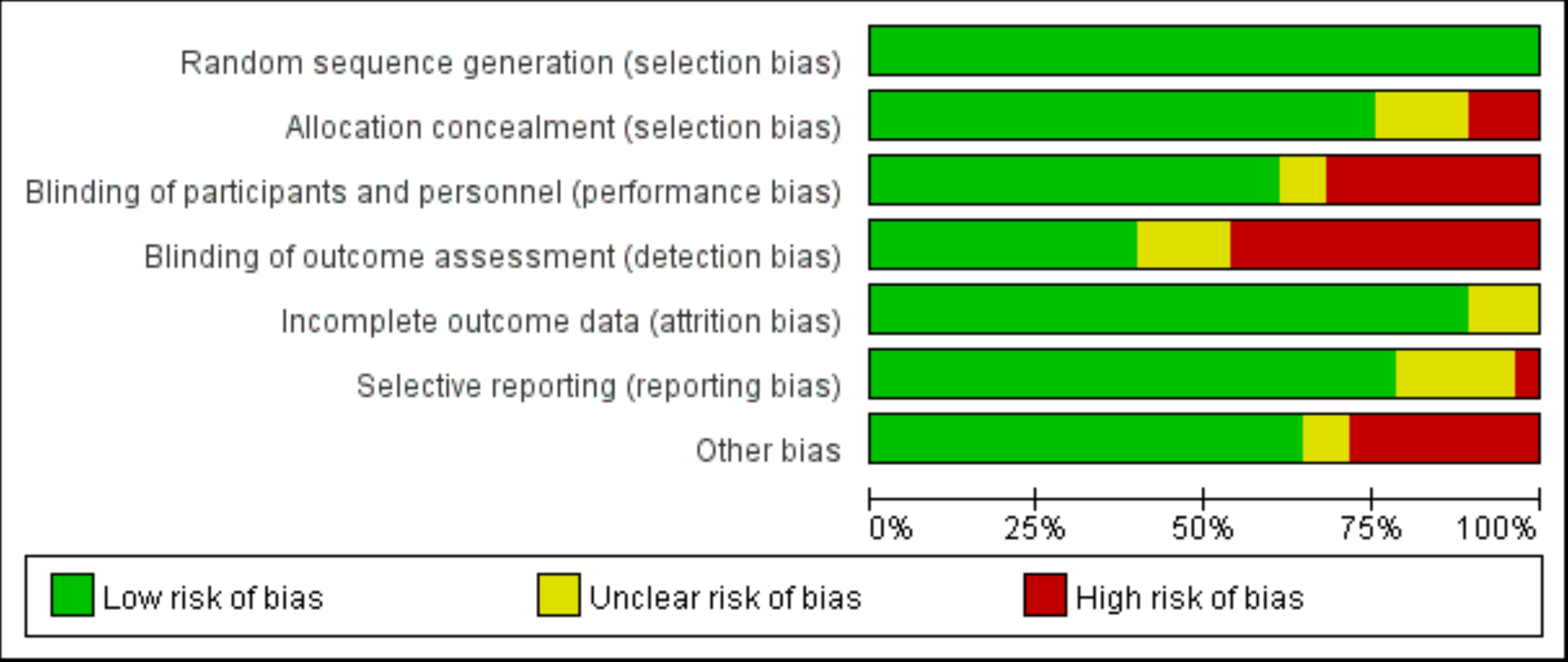


**B**

(A) Risk of bias summary: review authors' judgments about each risk of bias item for each included study. (B) Risk of bias graph: review authors' judgments about each risk of bias item presented as percentages across all included studies.

**Appendix Figure 2. Funnel plot for publication bias on state anxiety.**

**Appendix Figure 3. Funnel plot for publication bias on trait anxiety.**

**Appendix Figure 4. Funnel plot for publication bias on stress symptom.**

**Appendix Figure 5. Funnel plot for publication bias on sleep symptom.**

**Appendix Figure 6. The forest plot of the sensitivity analysis of Rosa damascene aromatherapy on overall anxiety.**

**
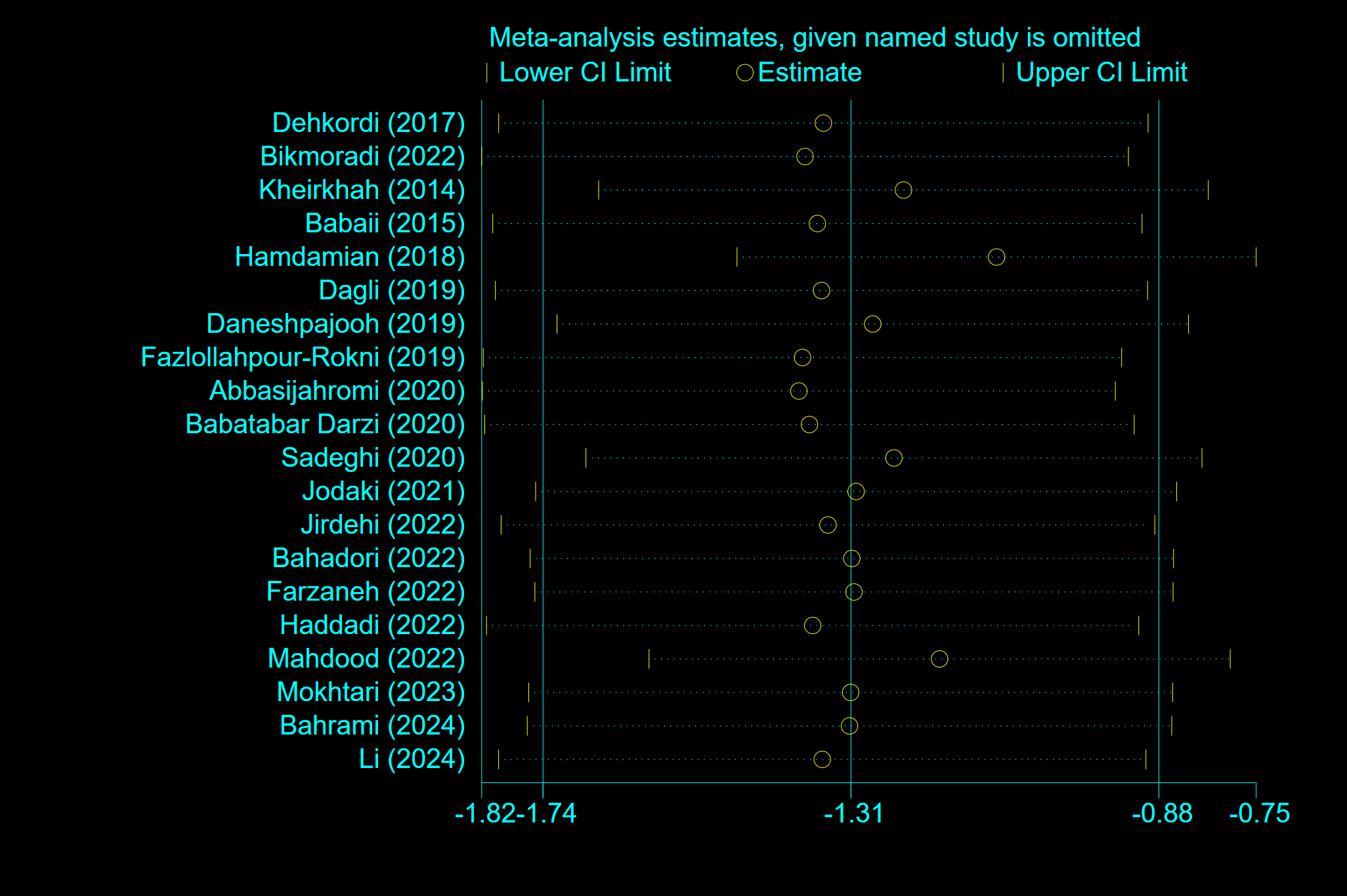
**

**Appendix Figure 7. The forest plot of the sensitivity analysis of Rosa damascene aromatherapy on state anxiety.**

**
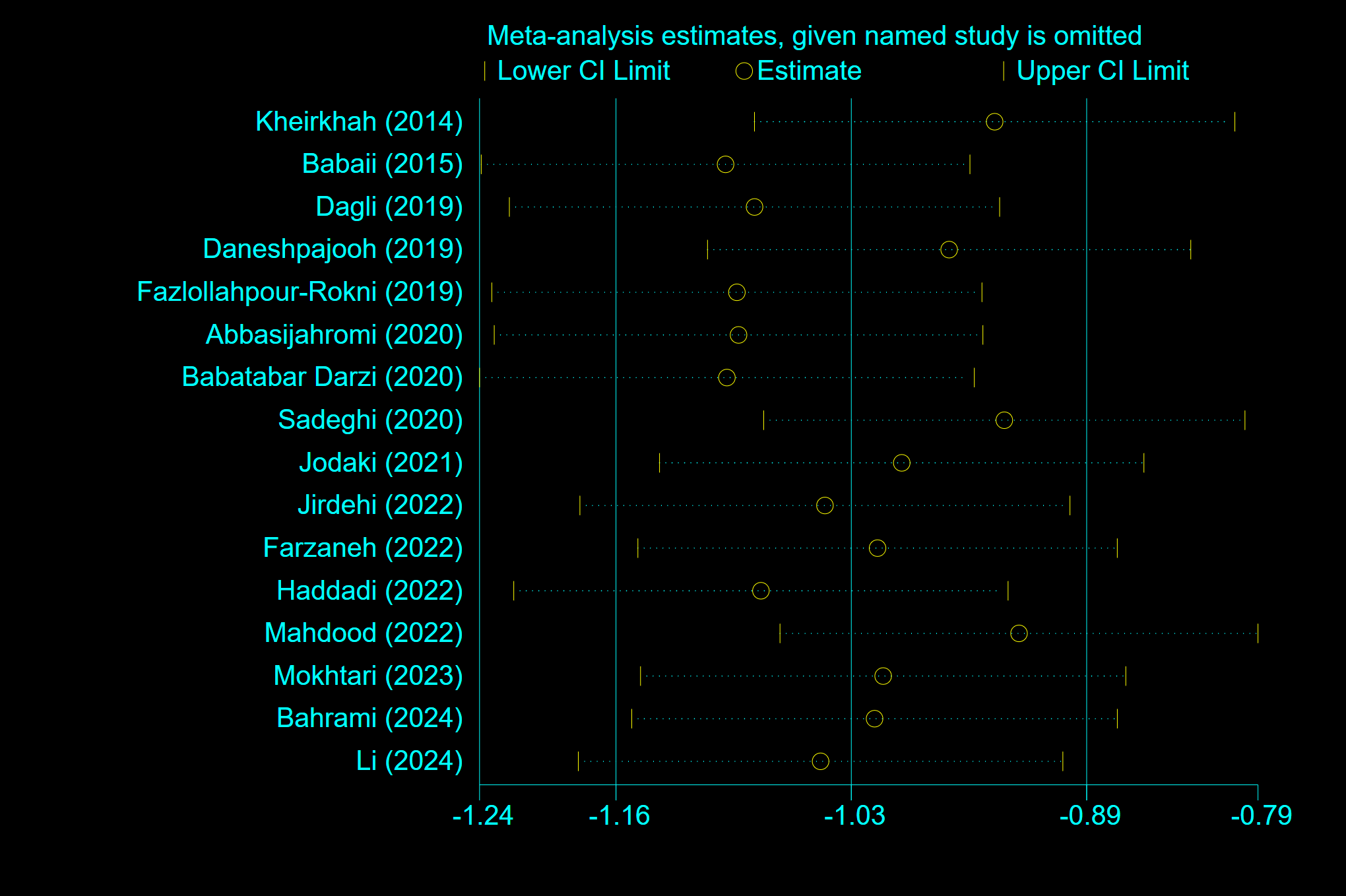
**

**Appendix Figure 8. The forest plot of the sensitivity analysis of Rosa damascene aromatherapy on trait anxiety.**

**
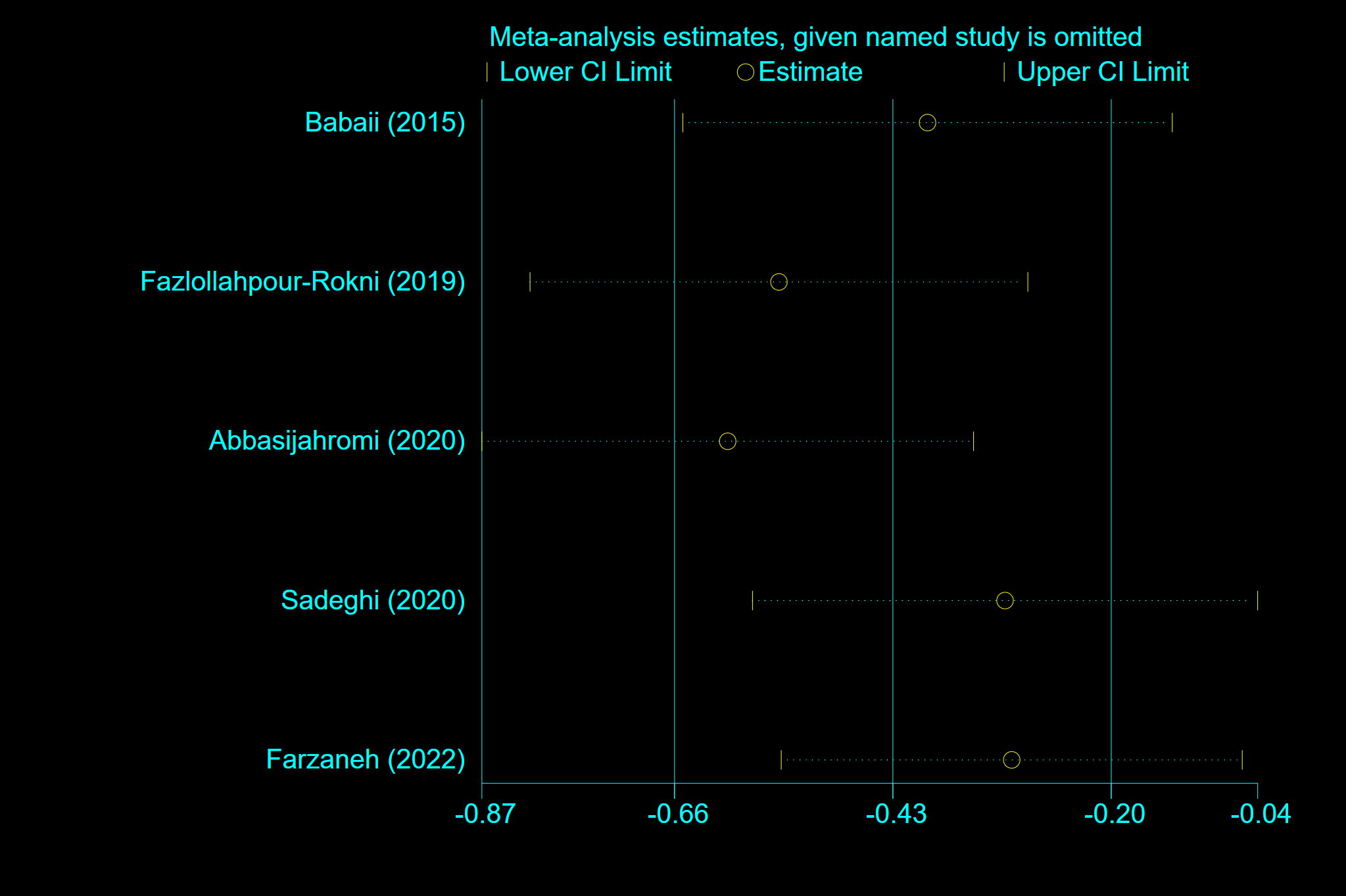
**

**Appendix Figure 9. The forest plot of the sensitivity analysis of Rosa damascene aromatherapy on stress symptom.**

**
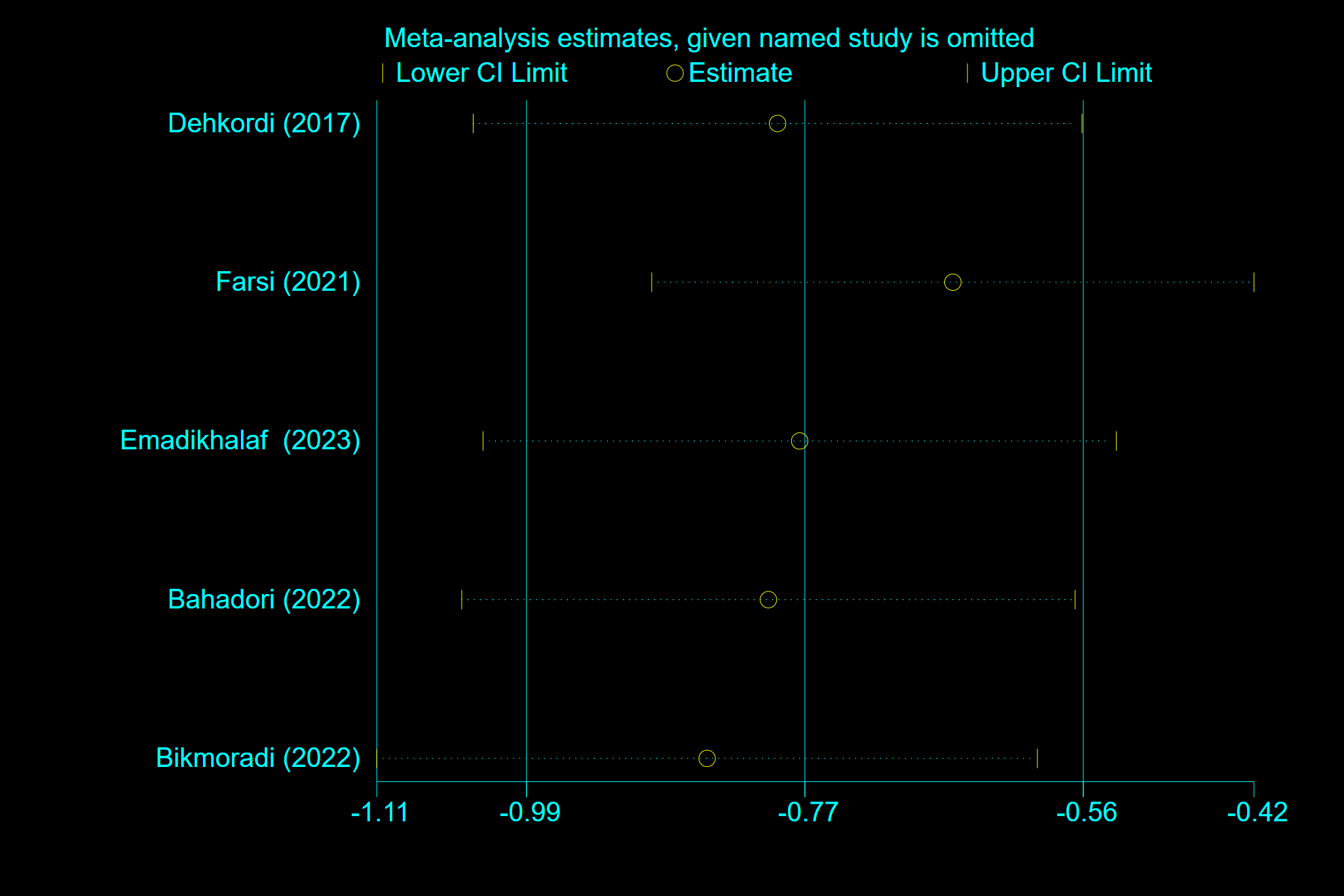
**

**Appendix Figure 10. The forest plot of the sensitivity analysis of Rosa damascene aromatherapy on sleep symptom.**


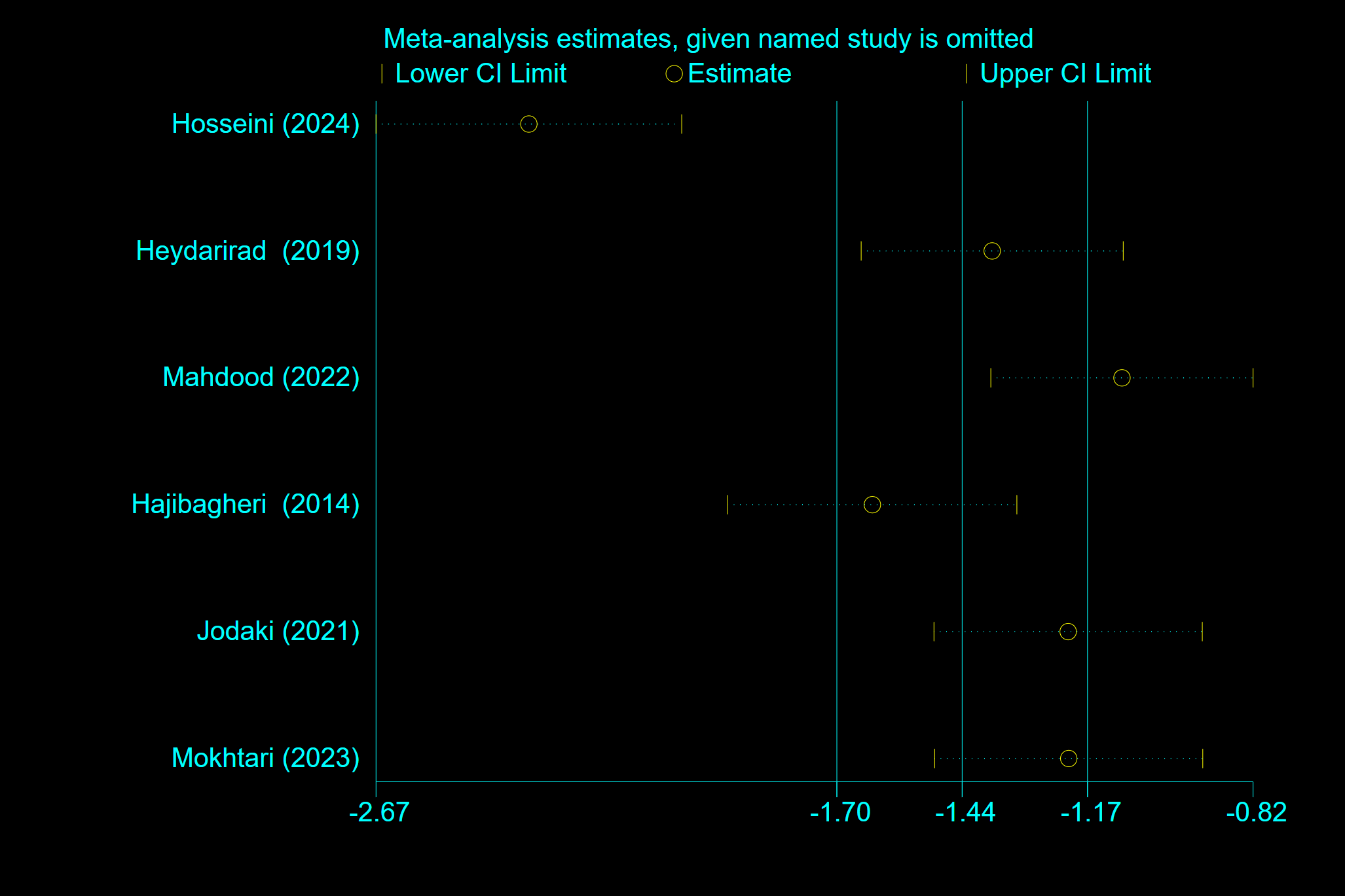

Supplement: Supplementary file 1 [file Supplementary_Material.DOCX]
